# Perceived Life Expectancy Among Dialysis Recipients: A Scoping Review

**DOI:** 10.1016/j.xkme.2023.100687

**Published:** 2023-06-14

**Authors:** Hannah Beckwith, Amarpreet Thind, Edwina A. Brown

**Affiliations:** 1 Renal Department, Imperial College Healthcare NHS Trust, London, United Kingdom; 2 Department of Renal Medicine, Imperial College London, London, United Kingdom

**Keywords:** Prognosis expectations, perceived life expectancy, treatment choices, prognostic uncertainty, shared decision-making, hemodialysis, dialysis, renal replacement therapy

## Abstract

**Rationale & Objective:**

Greater prognostic understanding is associated with higher quality care at the end of life. We undertook a scoping review to explore how long dialysis recipients expect to live.

**Study Design:**

Scoping Review

**Setting & Study Populations:**

People with kidney failure over 18 years old.

**Search Strategy & Sources:**

Studies were identified by searching Medline, Embase, APA PsycINFO, HMIC, and ProQuest database for terms related to “life expectancy”, “self-estimated”, and “end stage kidney disease”.

**Data Extraction:**

Search strategies reported 349 unique, potentially eligible studies, with 8 studies meeting the inclusion criteria after screening.

**Results:**

Significant mismatches between dialysis recipients and their health care provider’s estimations of prognosis were reported, with patients predicting significantly higher life expectancies than health care professionals and almost no agreement between patient and nephrologist’s estimates of 1-year survival. Documented cognitive impairment did not affect 1-year or 5-year prognosis estimates, nor did gender, age, time on dialysis, or discussing perceived life expectancy. Dialysis recipients who thought they were on the transplant list or who self-identified as African American reported higher perceived life expectancy, whereas people who were 75 years or older, or with fair or poor self-reported health status reported a lower perceived life expectancy. Those with a lower perceived life expectancy preferred care focusing on relieving pain and discomfort, whereas people who thought they had a higher chance of survival were significantly more likely to prefer life-extending care.

**Limitations:**

There is a marked paucity of research in this area, with most studies conducted in North American cohorts.

**Conclusions:**

Optimistic patient prognostic expectations persist in dialysis recipients. Given the effects of perceived life expectancy on treatment choices and subsequent quality of life, it is important that transparent discussions regarding prognosis are conducted with people receiving dialysis and their families.

**Plain-Language Summary:**

Understanding illness severity and prognosis allows people to make decisions and prioritize areas of their lives that are important to them. We undertook a scoping review to explore how long dialysis recipients expect to live. We found significant mismatches between the perceived life expectancy of people treated with dialysis and their health care providers. Perceived life expectancy influenced treatment choices; thus, those who thought they would die sooner prioritized care focusing on relieving pain and discomfort. Those who thought they had a higher chance of survival were more likely to prefer life-extending care (with potential effects on quality of life). It is important to have frank discussions about prognosis with people receiving dialysis, to empower individuals and help them make informed decisions about their care.

Patient-clinician communication underpins all areas of medicine. When done well, it can improve illness experience,^1^ mental health,^2^ patient satisfaction,^3^ recall, and understanding of information.^4^ Similarly, when done poorly, it can negatively affect interpersonal relationships^4^ and patient health outcomes.^1^ Sharing prognosis information has traditionally been viewed as one of the more challenging areas of communication with patients, and clinicians have highlighted numerous potential barriers. Sufficient time is needed within the clinical encounter and cultural and linguistic difficulties that can hinder open conversations, which some health care professionals find uncomfortable, because of lack of formal communication skills training.^5^, ^6^, ^7^, ^8^

But, most patients want to know their prognoses and their options for end-of-life care.^9^^,^^10^ Moreover, greater prognostic understanding is associated with decreased preferences for more intensive treatment^11^ and higher quality care at the end of life.^12^^,^^13^ It has been recognized that people with advanced chronic kidney disease (CKD) tend to experience less high quality end-of life care and receive more aggressive care compared with people with other noncancer diagnoses (such as those with heart failure or dementia), suggesting that prognostic understanding among people with advanced CKD is perhaps lower.^14^, ^15^, ^16^, ^17^ Furthermore, health literacy is often limited among older people with kidney failure, creating further barriers to achieving greater prognostic understanding.^18^ Individuals who do not have accurate prognostic awareness do not feel empowered to make informed decisions^19^; however, people with CKD have to make many decisions over their illness trajectory and across different care settings and sectors.^20^ Consequently, improving shared decision-making has been highlighted as a key patient research outcome priority within the nephrology community.^21^

Understanding the prognostic expectations of people with kidney failure has been an area of increased research interest over the last few years. There is an extensive literature base exploring prognostic expectations in oncology, but only more recently has this been investigated in people without cancer. Developing a better understanding of the prognostic expectations of people receiving dialysis is a key initial step in improving supportive and end-of-life care for nephrology patients. As such, we sought to undertake a scoping review to explore how long dialysis recipients expect to live.

## Methods

### Study Design

We chose to undertake a scoping review because this was an exploratory study. We wanted to identify the available literature^22^^,^^23^ and scoping reviews are particularly useful for examining emerging evidence.^24^^,^^25^ We used established guidance to inform search strategies, extraction, and synthesis of evidence.^24^^,^^26^ The PRISMA-ScR reporting tool was used to provide guidance on reporting of findings^26^ (Table S1).

### Search Strategy

Studies were identified by searching Medline (OVID), Embase, APA PsycINFO, Health Management Information Consortium, and ProQuest Database from the study’s inception to September 30, 2021. A manual search of relevant gray literature (ProQuest dissertations and Europe PMC) was also conducted. The protocol for search terms was piloted and reviewed by an external clinical librarian. Search terms related to life expectancy, self-estimated, and end stage kidney disease were used. Full details of the search strategy can be found in Item S1.

### Inclusion and Exclusion Criteria

Studies were included in which adults (aged ≥18 years) were asked to estimate how long they would live. Studies that included duration, or defined time periods, and chance or risk (for example, what is the chance you will be alive in 1 year) were included. When studies reported diseases other than end stage kidney disease (for example, heart failure and chronic obstructive pulmonary disease), data presented were assessed to see if individual diseases were reported separately and included only if kidney failure, or kidney failure with replacement therapy was distinctly reported. Studies were limited to those published in English only.

### Data Extraction (Selection and Coding)

Studies were selected independently by 2 reviewers, with disagreements resolved by discussion. This involved initial title and abstract screening, followed by full-text screening against the inclusion criteria. For relevant reviews, individual studies within the review were screened against the inclusion criteria. The software program Covidence was used to manage the process.^27^

### Assessment of Risk of Bias

The assessment of risk of bias was undertaken using the Risk of Bias Assessment tool for Nonrandomized Studies.^28^ This tool assesses 6 domains and ranks each with a high, low, or unclear risk of bias. The 6 domains are selection of participants (selection bias caused by the inadequate selection of participants), confounding variables (selection bias caused by the inadequate confirmation and consideration of confounding variables), measurement of exposure (performance bias caused by the inadequate measurement of exposure), blinding of outcome assessments (detection bias caused by the inadequate blinding of outcome assessments), incomplete outcome data (attrition bias caused by the inadequate handling of incomplete outcome data), and selective outcome reporting (reporting bias caused by the selective reporting of outcomes).^28^

## Results

**Figure 1:**
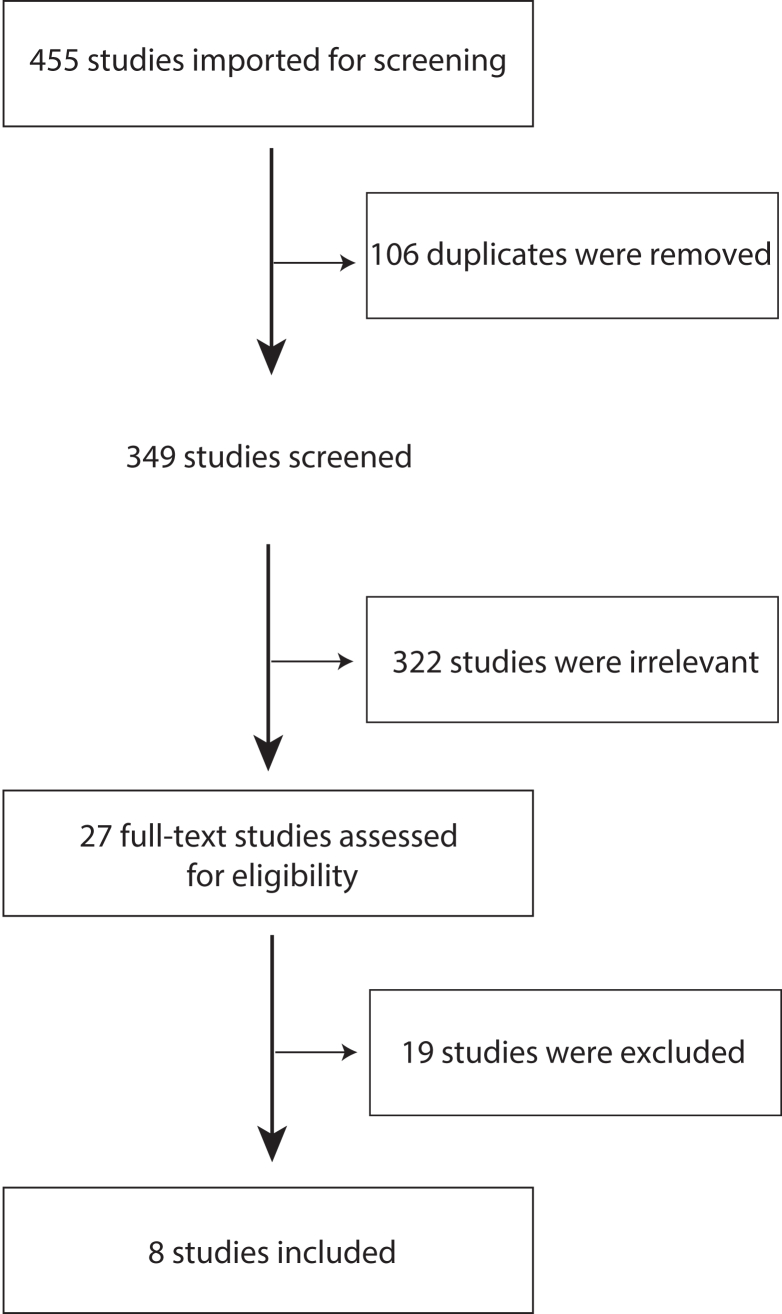
Flow chart for study inclusion criteria.

**Figure 2:**
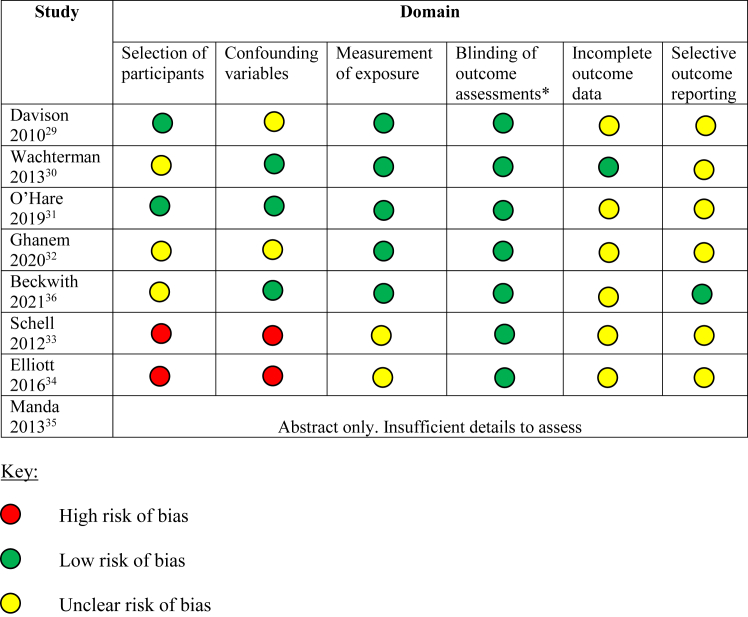
Risk of bias assessment per included study, the Risk of Bias Assessment tool for Nonrandomized Studies.^28^ ∗Given outcome assessments for this study were patient-reported (perceived life expectancy), a low risk of bias was assigned to all studies.

Search strategies reported 349 unique, potentially eligible studies, with 8 meeting the inclusion criteria after screening (Fig 1). Table 1 summarizes the characteristics of the included studies. Studies were published between 2010 and 2021 and were undertaken in Canada,^29^ the United States of America,^30^, ^31^, ^32^, ^33^, ^34^, ^35^, ^36^ and the United Kingdom.^33^ Six studies were quantitative^29^, ^30^, ^31^, ^32^, ^33^^,^^36^ and 2 studies were qualitative in nature.^34^^,^^35^ Fig 2 shows the risk of bias assessment per study.

**Table 1:** Patient Characteristics and Study Details

Study	Patient Group Studied and Setting	Cognitive Impairment	Race/Ethnicity	Study Design	Study Size
Davison et al^29^ (2010)	Patients with Stage 4 or 5 chronic kidney disease in a university-affiliated renal program in Canada (North Alberta)	Excluded	80.5% White, 7.2% Aboriginal, 8.1% Asian, 1.4% African American, and 2.4% other	Questionnaire-based study	584 patients: 238 non–dialysis-dependent (41%), 222 hemodialysis (38%), 73 peritoneal dialysis (12.5%), and 51 transplant (9%)
Wachterman et al^30^ (2013)	Patients receiving hemodialysis with estimated 1-y mortality risk of ≥20%. 2 community-based hemodialysis centers affiliated with tertiary medical care centers in the United States (Boston)	Excluded	19 (31%) White, 32 (52%) African American, 5 (8%) Asian, 1 (2%) other, and 5 (8%) not documented	Inperson interviews	62 patients receiving hemodialysis and 14 nephrologists
O’Hare et al^31^ (2019)	Patients at 31 dialysis facilities in the United States (Washington and Tennessee)	Included if cognitively able to provide informed consent	563 (56.7%) White, 268 (27.0%) African American, 83 (8.4%) Asian, 16 (1.6%) American Indian or Alaskan Native, 30 (3.0%) Native Hawaiian, or other Pacific Islander, 33 and (3.3%) other or missing	Questionnaire-based study	993 Patients: 988 patients receiving hemodialysis (99.5%), 5 patients receiving peritoneal dialysis (0.5%)
Ghanem et al^32^ (2020)	Patients receiving hemodialysis at a single dialysis center in North America (New York)	Excluded	49 (74%) White, 13 (20%) African American, and 4 (6%) other	Questionnaire-based study	66 patients receiving hemodialysis and 4 nephrologists
Beckwith et al^33^ (2021)	Patients receiving hemodialysis with estimated 1-y mortality risk of ≥20%. 3 hemodialysis centers affiliated with tertiary medical center in England (London)	Included	29 (57%) White, 16 (31%) Non-White, and 6 (12%) not documented	Structured interview (44/51, 86%) or mixed methods questionnaire (7/51, 14%)	51 Patients receiving hemodialysis, their named nurse and nephrologist.
Schell et al^34^ (2012)	Patients aged ≥65 y with stage 3-5 chronic kidney disease or receiving hemodialysis and nephrologists (note not necessarily the included patients’ nephrologists. 2 centers in the United States (North Carolina)	Excluded	11 (38%) White, and 18 (62%) African American	Qualitative semi-structured interviews	29 patients: 11 with chronic kidney disease (38%), 18 patients receiving hemodialysis (62%), and 11 nephrologists.
Elliott et al^35^ (2016)	Older dialysis patients (>70 y) and their family members at a single center in North America (Minnesota)	Excluded	27 (87%) White, and 4 (13%) not documented	Qualitative interview study	20 patients receiving dialysis (does not state if patients receiving hemodialysis or peritoneal dialysis) and 11 family members
Manda et al^36^ (2013) (abstract only)	Dialysis patients at 2 community dialysis centers in North America (Massachusetts)	Does not specify	Does not specify	Prospective, nonrandomized, mixed methods study	37 patients (does not state if patients receiving hemodialysis or peritoneal dialysis)

### Perceived Life Expectancy

Optimistic patient prognostic expectations were reported by most studies. Quantitatively, when participants were asked about their chance of survival over the next 12 months, 81% felt they had a 100% chance of survival,^32^ and 81% thought they had a 90% chance of survival^30^ (both studies were undertaken in the United States). By contrast, only 37% of the participants thought they had a 95% chance of 1-year survival in the United Kingdom.^33^ Similarly, when asked about their chance of survival over the next 5 years, in the US studies, 67%^32^ and 42%^30^ thought they had a 90% chance of living for 5 years or more, compared with 25% of the participants predicting a 95% chance of survival at 5 years in the United Kingdom.^33^

Significant mismatches between patient and provider estimations of prognosis were reported, with people receiving dialysis predicting significantly higher life expectancies than health care professionals and almost no agreement between patient and nephrologist estimates.^30^^,^^32^^,^^33^ One study compared the prognosis predictions of a nephrologist and a named hemodialysis nurse and found no difference between the 2 estimates.^33^ Two studies included only seriously unwell participants (≥20% 1-year mortality risk).^30^^,^^33^ Notwithstanding, the findings from all studies were very similar, suggesting that there is a marked lack of prognostic understanding among dialysis recipients.

It is not possible to know exactly how many prognostic expectations differed between nephrologists and people receiving dialysis owing to differences in study reporting. Three of the included studies directly examined prognostic discordance.^30^^,^^32^^,^^33^ Two studies took similar approaches to analysis, comparing patient with a nephrologist or health care professional and dividing them into 5 groups depending on the estimate of survival (%).^30^^,^^33^ Reporting methods from 1 study enabled discordant pairs to be easily identified (80% of pairs when estimating 1-year survival, 70% of pairs when estimating 5-year survival).^33^ The second study used a different scale for patient and nephrologist responses, meaning direct comparison was not possible.^30^ Both of these studies reported grouped outcomes as opposed to individual differences.^30^^,^^33^ The third study reported only when >20% prognostic discordance was present.^32^ Overall, nephrologists and health care professionals appeared to overestimate, and patients underestimate mortality risk when compared with the actual outcomes^30^^,^^33^ with patient estimates closer to survival rates.

Alongside optimistic prognostic expectations, significant optimistic transplant discordance was also noted, such as in the seriously unwell.^30^^,^^33^ Despite very few participants being listed for transplant, many felt that they were both suitable candidates or were listed on the transplant waiting list.

### Prognostic Uncertainty

Both people receiving dialysis and nephrologists reported uncertainty about how the disease would progress, which hindered open conversations about prognosis. As a result, nephrologists “generally do not discuss prognosis and the future unless prompted, either by the patient or in the setting of acute illness”^34^ This finding was supported by quantitative studies; in 1 study 0% and in another 53% of the participants reported that their nephrologists had discussed prognosis with them.^30^^,^^32^ Challenges to engaging in prognosis conversations were described, particularly, an “inability to predict the patient’s (disease) course,” and “concerns that discussions would be perceived as negative and remove patient’s hope.”^34^

### Desire to Know Prognosis

The reported desire to know the prognosis was variable. In 1 study, 90% of the people with kidney failure wanted to know detailed information about their medical condition, such as their prognosis.^29^ This contrasts with only 54% wishing to know the prognosis in a second study,^36^ and 47% specified they actively did not want to discuss prognosis with their nephrologist in a third study.^32^ Similarly, 76% of people receiving dialysis who had not already discussed end-of-life plans with a health care professional did not wish to explore this further.^33^ Two of the included studies were qualitative in nature, and so they examined this in more detail. One study, when exploring perceptions of “how (the) disease will progress” reported that “patients coped through avoidance and false hope,”^34^ which might explain the high numbers of participants not wanting to discuss prognosis in detail. The other found that perceptions of prognosis were “gained from experiential learning at the dialysis center and own experience, not from conversations with health care professionals,”^35^ which may also affect desire to engage in discussions of prognosis with nephrologists. One study reported that although 54% of the participants wanted to learn about their prognosis, only 62% knew what the term prognosis meant,^36^ highlighting that limited health literacy may be a significant contributory factor to people’s willingness to engage in these discussions.^18^

### Factors Influencing Perceived Life Expectancy

Documented cognitive impairment did not affect 1-year or 5-year prognosis estimates (although in many studies, people with cognitive impairment were excluded, Table 1) nor did self-reported gender, age, or time on dialysis.^33^ However, people receiving dialysis who thought they were on the transplant list were particularly confident they would be alive at both 1-year and 5-year.^33^ Discussing end-of-life care plans reported no effect on perceived life expectancy.^33^ Dialysis recipients who were aged ≥75 years or older, or had fair or poor self-reported health status were less likely to have a prognostic expectation of >10 years, whereas those who self-identified as African American were more likely.^31^

### Interplay between Perceived Life Expectancy and Treatment Choices

Multiple studies explored the interplay between prognostic expectations and treatment choices. Most participants did not want life-extending treatment at the expense of prolonging pain and discomfort.^29^, ^30^, ^31^, ^32^ However, those with a lower perceived life expectancy preferred care focused on relieving pain and discomfort,^33^ and those who thought they had a higher chance of survival were particularly more likely to prefer life-extending care.^30^^,^^37^ People receiving dialysis who were more optimistic than their nephrologist about transplant likelihood were also more likely to report a preference for life-extending care.^30^

## Discussion

In a scoping review, we identified 7 full studies and 1 additional abstract exploring perceived life expectancy among dialysis recipients, with most being published in recent years. This illustrates the increased recognition of importance of conducting research in this field and the developments in routine advance care planning in practice. Thus far, studies undertaken have been mostly conducted in North America. One study has been conducted in the United Kingdom, but further research in Europe, Africa, Asia, Australia, or Oceania is urgently needed.

People receiving dialysis have optimistic expectations of life expectancy and transplant suitability. This is not unique to people with kidney disease indeed, similar studies have shown optimistic prognostic expectations in people with advanced cancer, heart failure, and chronic obstructive pulmonary disease.^38^, ^39^, ^40^, ^41^ Similarly, included studies demonstrated a significant mismatch between patient perceptions of prognosis and those of their care providers. This is also regularly reported in oncology literature, even if prognostic discussions have occurred in the 3 months preceding the study.^42^ It is therefore critically important to have frank and open discussions surrounding the estimated prognosis and to check the patient’s and family’s understanding of the conversations.

Within nephrology, optimistic transplant discordance is associated with increased perceived 1-year and 5-year survival estimates.^33^ This may offer a unique opportunity for nephrologists to screen dialysis recipients for optimistic prognostic expectations. Thus, providing an opening point for discussion and facilitating deeper conversations about perceived life expectancy, treatment expectations, or advance care planning.

Both people receiving dialysis and nephrologists reported uncertainty about how the disease would progress, which hindered open conversations about life expectancy. Challenges to estimating accurate prognoses are well recognized.^43^ A seminal early article reporting the Study to Understand Prognoses and Preferences for Outcomes and Risks of Treatments described a randomized control trial providing physicians with computer generated prognostic estimates, to see whether this improved end-of-life care.^38^ Despite the generated prognostic estimates, <20% of physicians discussed prognostic information with their patients, suggesting a reticence among physicians to facilitate discussions around prognosis even when estimates were available. Similarly, another study found that when hospice patients requested survival estimates from physicians, they received them only 37% of the time.^44^ This is concerning when a lack of prognostic understanding may impede treatment decisions, and result in later referrals to, or underutilization of hospice care.^6^^,^^12^^,^^45^

This scoping review might suggest that people receiving dialysis in the United Kingdom have better prognostic understanding than those from North America, although only 1 study met inclusion criteria from the United Kingdom. The underlying reasons for this are not clear. At a systems level, differences in palliative care infrastructure and funding between the 2 countries may underpin some of the differences seen.^46^^,^^47^ However, intercenter variation was also noted,^33^ so differences in prognostic understanding may also reflect more the communication and influence of individual practicing physicians.

The reported desire to know the prognosis in the included studies was very variable. Again, the reasons for these variations are unclear. Within the oncology evidence-base, people with terminal cancer have identified a sense of ambiguity regarding prognostic information; that is, they want to be told, but simultaneously do not want to know.^48^

However, one of the included studies (abstract only) reported that although 54% of the participants wanted to learn about their prognosis, only 62% knew what the term prognosis meant,^36^ highlighting the importance of checking patient understanding. Other studies have suggested that understanding of terminology used in end-of-life conversations among people receiving dialysis is even lower.^18^ Limited health literacy is common in all stages of CKD, with a meta-analysis reporting a median prevalence of 23%,^49^ reiterating the importance of not only having open prognostic discussions but checking and confirming understanding.

Very few factors appear to influence perceived life expectancy among dialysis recipients. Predictors of reporting a low perceived life expectancy among adults without kidney failure included older age, male sex, and having a diagnosis of cancer or diabetes.^50^ This contrasts with the findings of this study, in which self-reported gender and age showed no significant influence on perceived life expectancy. A low sense of control over life, low satisfaction with life, and worse self-reported health were associated with low perceived life expectancy among older adults without kidney failure,^50^ whereas poor self-reported health status was found to have an effect on people with kidney failure.^51^^,^^52^ This highlights the complex interplay between perceived health and life expectancy.

This study found those with a lower perceived life expectancy preferred care focusing on relieving pain and discomfort,^33^ and those who thought they had a higher chance of survival were significantly more likely to prefer life-extending care.^30^^,^^37^ People receiving dialysis who were more optimistic than their nephrologist about transplant likelihood were also more likely to report a preference for life-extending care.^30^ The influence of perceived life expectancy on treatment choices has also been explored in people with lung, colon, bladder, and breast cancer.^11^^,^^53^^,^^54^ Similar to the results from this study, people with advanced cancer who thought they were going to live longer were more likely to favor life-extending and aggressive therapy over comfort care.^11^

Discordance between prognostic estimations of dialysis recipients and physicians is important because disparities between patients and clinicians can be associated with negative outcomes such as reduced treatment adherence, higher rates of hospitalization, and lower hospice use.^55^, ^56^, ^57^ Furthermore, recognizing that pessimistic estimations of life expectancy can negatively affect the quality of life of both people with cancer and their caregivers.^58^ Oncology services have reported successful interventions, such as the provision of psychosocial support to improve the wellbeing of people with limited life expectancy.^59^^,^^60^ Studies are also currently in progress to evaluate the efficacy of communication support programs, given the challenges of communicating uncertainty about prognoses.^61^ Similar findings have been reported in nephrology, with nephrologists choosing to avoid end-of-life discussions and an absence of formal training in how to communicate prognostic uncertainty.^8^^,^^62^ Thus, formal communication skills training for nephrologists in advance care planning could be beneficial.

A major strength of this study is the use of the scoping method, which has allowed us to present a broad overview of available literature and to present, compare, and contrast both qualitative and quantitative research. The main limitation of this study is the paucity of research in this area, lack of available literature, and bias toward North American studies. Despite this, several key findings have been identified and highlighted (Box 1) and will act as a starting point for future research.

Box 1 **Key Messages**

• This scoping review explores how long dialysis recipients expect to live. We found the following:
  ⋄ Optimistic prognostic expectations among people receiving dialysis.
  ⋄ Almost no agreement between the patients’ and the nephrologists’ estimates of 1-year survival.
  ⋄ Perceived life expectancy influenced by treatment choices.
• We suggest that transparent discussions regarding prognosis should be conducted with people receiving dialysis and their families.

In conclusion, optimistic patient prognostic expectations persist among dialysis recipients. Even when patients were selected for higher mortality risk, very few felt they had a reduced chance of survival, highlighting limited prognostic understanding. Given the effects of perceived life expectancy on treatment choices and subsequent quality of life, it is important that transparent discussions regarding prognosis are conducted with people receiving dialysis and their families.

## Supplementary Material

Supplementary File (PDF) Item S1; Table S1.
